# Comparative Genomics and Physiology of *Akkermansia muciniphila* Isolates from Human Intestine Reveal Specialized Mucosal Adaptation

**DOI:** 10.3390/microorganisms10081605

**Published:** 2022-08-09

**Authors:** Janneke P. Ouwerkerk, Hanne L. P. Tytgat, Janneke Elzinga, Jasper Koehorst, Pieter Van den Abbeele, Bernard Henrissat, Miguel Gueimonde, Patrice D. Cani, Tom Van de Wiele, Clara Belzer, Willem M. de Vos

**Affiliations:** 1 Laboratory of Microbiology, Wageningen University, 6708 WE Wageningen, The Netherlands; 2 Laboratory of Systems and Synthetic Biology, Wageningen University, 6708 WE Wageningen, The Netherlands; 3 Center for Microbial Ecology and Technology (CMET), Ghent University, 9000 Ghent, Belgium; 4 Department of Biotechnology and Biomedicine (DTU Bioengineering), Technical University of Denmark, 2800 Lyngby, Denmark; 5 Department of Biological Sciences, King Abdulaziz University, Jeddah 22254, Saudi Arabia; 6 Instituto de Productos Lácteos de Asturios (IPLA), Consejo Superior de Investigaciones Científicas (CSIC), 28006 Madrid, Spain; 7 Metabolism and Nutrition Research Group, Louvain Drug Research Institute, Walloon Excellence in Life Sciences and BIOtechnology (WELBIO), UCLouvain, Université Catholique de Louvain, 1348 Brussels, Belgium; 8 Human Microbiome Research Program, Faculty of Medicine, University of Helsinki, 00100 Helsinki, Finland

**Keywords:** *Akkermansia muciniphila*, *Akkermansia glycaniphila*, Verrucomicrobia, human isolates, comparative genomics, CAZyome

## Abstract

*Akkermansia muciniphila* is a champion of mucin degradation in the human gastrointestinal tract. Here, we report the isolation of six novel strains from healthy human donors and their genomic, proteomic and physiological characterization in comparison to the type-strains *A. muciniphila* Muc^T^ and *A. glycaniphila* Pyt^T^. Complete genome sequencing revealed that, despite their large genomic similarity (>97.6%), the novel isolates clustered into two distinct subspecies of *A. muciniphila*: Amuc1, which includes the type-strain Muc^T^, and AmucU, a cluster of unassigned strains that have not yet been well characterized. CRISPR analysis showed all strains to be unique and confirmed that single healthy subjects can carry more than one *A. muciniphila* strain. Mucin degradation pathways were strongly conserved amongst all isolates, illustrating the exemplary niche adaptation of *A. muciniphila* to the mucin interface. This was confirmed by analysis of the predicted glycoside hydrolase profiles and supported by comparing the proteomes of *A. muciniphila* strain H2, belonging to the AmucU cluster, to Muc^T^ and *A. glycaniphila* Pyt^T^ (including 610 and 727 proteins, respectively). While some intrinsic resistance was observed among the *A. muciniphila* straind, none of these seem to pose strain-specific risks in terms of their antibiotic resistance patterns nor a significant risk for the horizontal transfer of antibiotic resistance determinants, opening the way to apply the type-strain Muc^T^ or these new *A. muciniphila* strains as next generation beneficial microbes.

## 1. Introduction

Our knowledge on the composition, functionality and importance of the gastrointestinal (GI) microbiota has grown exponentially over the past decades. The host benefits in various ways from the presence of microbes as they degrade our food, produce short chain fatty acids (SCFAs), synthesize vitamins and produce a range of other metabolites [1,2]. Beyond the metabolism of food components, the microbiota also plays an indispensable role in stimulating the immune system, strengthening the epithelial barrier, inhibiting pathogen colonization and regulating many more key physiological processes [2,3].

*Akkermansia muciniphila* is one of the key microbial species residing at the mucosal microbiota–host interface. A distinguishing characteristic of the type-strain Muc^T^ is its ability grow rapidly on human mucin and use this glycoprotein as a sole carbon, energy and nitrogen source, making it a mucin specialist [4]. In mouse experiments, *A. muciniphila* was shown to be among the first users of the labelled mucin glycans in the colon [5]. Furthermore, *A. muciniphila* Muc^T^ was found to actively stimulate host mucin production, thus increasing mucus layer thickness, in mice and improving gut-barrier function both in mouse gut and human cell lines [6]. Together with other primary mucin degraders, *A. muciniphila* forms the mucosal microbiota, specialized in partially degrading mucin glycans and producing SCFAs, both which are utilized by secondary colonizers for growth [7,8].

Various studies have shown that *A. muciniphila* is associated with a healthy GI tract, with its abundance inversely correlating with several pathologies, such as obesity, type 1 and type 2 diabetes, inflammatory bowel disease and appendicitis [6,9,10], for a recent review see [11]. The abundance of *A. muciniphila* may be even used as a prognostic marker for the success of dietary interventions of diabetes [12]. Germ-free mice mono-associated with *A. muciniphila* Muc^T^ showed metabolic and immune signaling specifically in the colon [13]. Moreover, administration of *A. muciniphila* Muc^T^ cells in obese mice reversed high-fat-diet-induced metabolic disorders, including fat-mass gain, metabolic endotoxemia and insulin resistance [6]. Given the shown immunostimulatory and barrier-strengthening capacities of *A. muciniphila* [13,14,15], administration of *A. muciniphila* has been suggested as a novel treatment for obesity and its associated disorders [16]. This potential was found with live, as well as pasteurized, *A. muciniphila* Muc^T^ cells in obese and diabetic mice, [10,17] and, importantly, this could be replicated in an intervention study in humans with overweight, obesity and metabolic syndrome [18].

The vast majority of intervention studies have been performed using the *A. muciniphila* type-strain Muc^T^, which was isolated from a healthy adult and found to grow optimally in mucin-based medium [4,11]. The 2.7-Mb genome of this strain encodes a complex mucin degrading capacity, as 61 secreted proteins were predicted to be involved in mucin degradation [19]. A recent survey of 2420 metagenomes of *Akkermansia* spp. from human and animals showed that all except the one from *A. glycaniphila* type-strain Pyt^T^ isolated previously from the python [20,21], showed identical 16S rRNA sequences that could be grouped into five distinct phylogenetic clusters, Amuc1, Amuc2, Amuc3 and Amuc4, while some unassigned strains grouped into a cluster, designated AmucU here [22].

Here, we describe the isolation of six new *A. muciniphila* strains originating from the human GI tract and described their genomic and physiological divergence in comparison with *A. glycaniphila*. All isolates showed highly conserved overall coding capacity and mucus specialization. Genome sequence analysis revealed the stratification of the *A. muciniphila* isolates into two groups differing in genomic regions, with extensive conservation of mucin degradation pathways, illustrating their niche adaptation to the mucus interface. We further investigated the carbohydrate-active enzymes (CAZymes) profiles of all isolates in comparison to that of the type-strains to showcase their glycan utilization potential and performed a detailed analysis of the proteome of two of these. Finally, we also determined their antimicrobial resistance profiles of the newly isolated strains in comparison with that of *A. muciniphila* Muc^T^ and *A. glycaniphila* Pyt^T^, as to deduce the intrinsic insensitivity to clinically used antibiotics, since this is of relevance for their application as next generation beneficial microbes [11,23,24].

## 2. Materials and Methods

### 2.1. Isolation of Akkermansia muciniphila Isolates and Growth Conditions

Strains H1, H2, H3 and H4 were isolated from fresh samples taken from the M-SHIME (Mucosal Simulator of the Human Intestinal Microbial Ecology) mucosal beads, initially inoculated with fecal samples from single healthy individuals. The experimental details are described in [25]. Strains H1 and H2 derived from two different reactors seeded with subject A and B, respectively. Strains H3 and H4 were isolated from the same reactor seeded with subject C. The H5 and H6 strains were also isolated from the same healthy subject D who donated a fresh fecal sample, which was immediately resuspended in anaerobic PBS (pH 7) containing 0.5 g/L cysteine-HCl (Sigma-Aldrich, St. Louis, USA). The subjects were all in between 24–35 years of age and healthy with a normal BMI (Table S1).

Both M-SHIME and fecal samples were diluted tenfold in anaerobic mucin medium, composed of a bicarbonate-buffered basal medium (pH 6.5–7) supplemented with 0.5% (*v/v*) purified and dialyzed porcine gastric mucin (Type III, Sigma-Aldrich) [4,26]. All samples were incubated for 24 h at 37 °C in 30 mL serum bottles, containing 10 mL mucin media (see above), sealed with butyl rubber stoppers under anaerobic conditions (1.5 atm N_2_/CO_2_ (80:20 *v*/*v*). Enrichment of strains was achieved by repeated serial dilutions. Strains were then further purified by repeated plating of single colonies on anaerobic mucin medium agar (0.8% *w*/*v*, Bacto Agar, BD, Franklin Lakes, NJ, USA). Purified strains were stored in mucin medium containing glycerol (25% *v*/*v*) at −80 °C.

### 2.2. Assessment of Sugar Fermentation Capacity

All strains were grown on mucin-supplemented medium (see above). For growth on other C-sources, the bicarbonate-buffered basal medium was supplemented with 16 g/L tryptone (Oxoid, Sigma-Aldrich, St. Louis, MO, USA) and either galactose, mannose, maltose, lactose, fucose, N-acetylglucosamine or N-acetylgalactosamine (10 mM final concentration) or glucose (25 mM final concentration). Growth was monitored by measuring optical density at 600 nm (Ultraspec 10, Biosciences, Biochrom, Cambridge, UK). Substrate conversion and product formation were measured by HPLC as previously described [27].

### 2.3. Proteome Analysis

*A. muciniphila* and *A. glycaniphila* strains were grown as described in Section 2.2, except that basal medium was supplemented with 20 g/L tryptone, 4 g/L threonine, 12.5 mM N-acetylglucosamine, 13.8 mM glucose. Bacteria were grown for approximately 30 h, after which pellets were harvested by centrifugation for 15 min at >12,000× *g*. Pellets were washed in PBS, and spun down again, after which they were resuspended in PBS with cOmplete™ Protease Inhibitor Cocktail (1 tablet in 50 mL, Merck, St. Louis, MO, USA). Next, suspensions were sonicated until clear with a Bandelin Sonopuls HD equipped with a MS 72 microtip, with an amplitude of 30% and repeated sonication (1 s ON–2 s OFF). Remaining cell debris was removed by filtration. Suspensions were kept on ice as much as possible. Experiments were performed in single replicates per bacterial strain. Proteomic analysis was performed at the Functional Genomics Center Zurich by LC-MS/MS try and proteins were identified using Mascot. The 100 highest expressed proteins of both Pyt^T^ and H2 were selected and blasted against the genome of Muc^T^ using the Protein Basic Local Alignment Search Tool (pBLAST) of NCBI to retrieve RefSeq Proteins. All hits were translated to UniProtKB or—if not available—UniParc IDs using UniProt. The highest annotated hit was selected per protein and if not available, UniParc IDs were given (Supplementary File).

### 2.4. Antibiotic Resistance Profiles

*A. muciniphila* strains were streaked on GAM (Gifu Anaerobic Medium) agar (Nissui Pharmaceutical Co., Ltd., Tokyo, Japan) to assure purity and further grown in pre-reduced GAM medium in anaerobic conditions at 37 °C.

Antibiotic resistance tests were performed following EFSA (European Food Safety Authority, Parma, Italy) guidelines on assessment of bacterial susceptibility to antimicrobials of human and veterinary importance [28], indications for anaerobic bacteria from the Clinical and Laboratory Standards Institute (CLSI) and the ISO 10932:2010 standard for bifidobacterial and non-enterococcal acid lactic bacteria. These standards follow the microdilution method from colonies using antibiotic-precoated plates: either the ANO2B (Sensititre^TM^ Anaerobe plate format, Thermo-Fisher Scientific, St. Louis, USA) or the VetMIC Lact-1 and Lact-2 (SVA National Veterinary Institute Sweden, Uppsala, Sweden).

*Bacteroides fragilis* ATCC 25285 was included as a quality control in the Sensititre plates. This strain was grown in Brucella agar supplemented with hemin (5 μg/mL), vitamin K1 (1 μg/mL) and lysed horse blood (5%) (Sigma-Aldrich, St. Louis, MO, USA). A standardized inoculum was prepared by suspending colonies in 0.85% NaCl solution to achieve an OD_600_ of 0.6 (i.e., equivalent of 2 × 10^8^ CFU/mL). An amount of 100 μL of a 1:100 further dilution in Brucella supplemented broth of this start inoculum were plated on the ANO2B plates and incubated for 48 h.

For the assessment using the VetMIC plates, *Lacticaseibacillus paracasei* ATCC 334 was used as a positive control. A standardized inoculum in 0.85% NaCl of OD_600_ 0.6 (i.e., 2 × 10^8^ CFU/mL) was used to inoculate LSM broth (90% Isosensitest broth (Oxoid) and 10% MRS broth (Merck, St. Louis, MO, USA)) at 1:1000. 100 μL of each bacterial suspension was used to inoculate the wells of the VetMIC plates.

The *Akkermansia* strains were propagated in GAM broth and anaerobically incubated at 37 °C under a 10% (*v*/*v*) H_2_ and 80% (*v*/*v*) N_2_ atmosphere. A suspension of OD_600_ 0.5–0.6 was prepared in 0.85% NaCl. 100 μL of a further dilution of 1:100 in GAM broth was then distributed in the wells of the ANO2B, whilst the VetMIC Plates were incubated with 100 μL of a 1:1000 dilution.

Plates were incubated under anaerobic conditions at 37 °C and followed at the second, fourth and seventh day. The Minimum Inhibitory Concentration (MIC) was defined as the lowest antibiotic concentration at which there was no visual growth (ISO 10932:2010) and determined at 1-week incubation time.

### 2.5. 16S rRNA Gene Sequence Identification

DNA obtained from the pure cultures was amplified with universal primers 27F (AGAGTTTGATCMTGGCTCAG) and 1492R (CGGCTACCTTGTTACGAC) as previously described [29]. In order to obtain an almost complete 16S sequence, the amplicons were purified using a high pure PCR cleanup micro kit (Roche Diagnostics, St. Louis, MO, USA), ligated into the pGEMTeasy vector system (Promega, Madison, USA) and transformed into *E. coli* XL1-blue competent cells (Agilent Technologies, Santa Clara, CA, USA) following manufacturer’s instructions. Five transformants per isolated were selected, plasmids were extracted and their inserts sequenced (GATC Biotech, Ebersberg, Germany) using both flanking binding sites (T7 TATTTAGGTGACACTATAG and SP6: TAATACGACTCACTATAGGG). Vector, primers and low-quality ends of the sequences were trimmed, and both sides were assembled to obtain the full 16S rRNA sequence. To identify potential sequencing errors, the five complete sequences were aligned and checked using DNABaser sequence assembler v4. All sequences were aligned using SINA [30] and subsequently imported in ARB [31].

To identify potential other *Akkermansia* species, DNA was extracted from human fecal samples using the repeated bead-bead method as described in [32] and used as a template to detect Verrucomicrobia-specific 16S rRNA gene sequences by PCR. Primers used: VER_37: TGGCGGCGTGGWTAAGA and VER_673: TGCTACACCGWGAATTC [33]. The amplicons were cloned into the pGMTeasy vector and sequenced as described above.

### 2.6. Phylogenetic Tree Construction

The phylogenetic tree was built on *Akkermansia muciniphila* core genes using PhyloPhlAn3 with the flags “--force_nucleotides --trim greedy --fast --diversity low”. The genome set consists of isolate genomes as well as Metagenome-Assembled Genomes that were curated as described previously [22].

### 2.7. DNA Isolation and Genome Sequencing

High molecular weight genomic DNA was extracted from overnight-grown cultures as previously described [34]. DNA quality and concentrations were determined by spectrophotometric analysis using NanoDrop equipment (Thermo Scientific, St. Louis, MO, USA) and by electrophoresis on a 1% agarose gel. DNA was stored at −20 °C until subsequent sequencing.

Genome sequencing was carried out at the Institute of Biotechnology, University of Helsinki (Helsinki, Finland). A MiSeq library was generated and sequenced on an Illumina MiSeq Personal Sequencer with 250 bp paired-end reads and an insert size of 500 bp. Reads were assembled using Ray (k-mer 101) [35]. After initial comparison of the genomes, the genome of H2 was subsequently sequenced by single molecule sequencing using a PacBio RS II instrument. Assembly was performed with PacBio SMRT analysis pipeline v2.2 and the HGAP protocol [36]. Default settings were used except for the following settings: minimum sub-read length 500, minimum polymerase read length quality 0.80, minimum seed read length 7000, split target into chunks 1, alignment candidate per chunk 24, genome size 3,000,000, target coverage 30, overlapper error rate 0.06, overlapper mini length 40 and overlapper k-mer 14.

### 2.8. Genome Annotation

Annotation was carried out with an in-house pipeline consisting of Prodigal v2.5 for prediction of protein coding DNA sequences (CDS) [37], InterProScan 5RC7 for protein annotation [38], tRNAscan-SE v1.3.1 for prediction of tRNAs [39] and RNAmmer v1.2 for prediction of rRNAs [40]. Additional protein function predictions were derived via BLAST identifications against the UniRef50 [41] and Swissprot [42] databases. Subsequently, the annotation was further enhanced by adding EC numbers via PRIAM [43]. Non-coding RNAs were identified using rfam_scan.pl v1.04, on release 11.0 of the RFAM database [44]. CRISPRs (Clustered Regularly Interspaced Short Palindromic Repeats) were annotated using CRISPR Recognition Tool v1.1 [45]. Carbohydrate active-enzymes were identified using the procedure used for the updates of the CAZy database [46] A further step of automatic curation was performed by weighing the annotation of the different associated domains, penalizing uninformative functions (e.g., “Domain of unknown function”), and prioritizing functions of interest (e.g., domains containing “virus”, “bacteriophage”, “integrase” for bacteriophage related elements; similar procedure for different other functions). CRISPR location and orientation was predicted using CRISPRDirection [47] with high confidence. CRISPR repeats were used in CRISPRMap [48] to identify the CRISPR/Cas system, after using CRISPRFinder [49] to obtain the correct format. CRISPR spacer targets were identified using CRISPRTarget [50]. CRISPR spacers were BLASTed against each other with E-value threshold of 0.1 and visualized as bitscores using Circos [51,52]. Single Nucleotide Polymorphisms (SNPs) were detected using MUMmer software v3.23, commands “nucmer—maxmatch-c 500” and “show-snps-C’ [53].

### 2.9. Strain Identifiers and Accession Numbers

The isolated strains and the characteristics of their hosts are described in Table S1. Draft genomes (raw data and annotated assembly) of H1, H3, H4, H5 and H6 were deposited at the NCBI under project number PRJNA270907, while the closed genome of H2 was deposited with ACC number: CP010553. Similarly, the 16S rRNA sequences of the isolated strains were deposited with accession numbers KP418639-KP418644 while that of the cloned amplicons had accession numbers KP418645-KP18776.

### 2.10. Statistical Analyses

Growth data are presented as mean with standard deviation (SD). Differences between two groups were assessed using Student’s *t*-test with *p* < 0.05.

## 3. Results

### 3.1. Enrichment and Isolation of Six Novel Akkermansia Strains from Human Feces

Starting from in vitro cultured mucosal microbiota samples, originally derived from individual fecal samples received from four healthy human volunteers, six novel mucin-degrading anaerobes could be isolated (Table S1). Enrichment experiments included serial dilutions followed by incubation in anaerobic mucin medium. Finally, purification of single colonies resulted in the isolation of *Akkermansia*-like bacteria by identifying colonies based on previously described morphology characteristics [4] and reacting positively in an *Akkermansia*-specific colony PCR [54].

This procedure resulted in the isolation of six *Akkermansia* isolates (Table S1). All isolates were Gram-negative, non-motile, non-spore-forming, oval-shaped bacteria of approximately 0.5–1 µm that could grow singly, in pairs, and form aggregates in a mucin-based medium.

All new isolates could be classified as *A. muciniphila* strains based on their 16S rRNA sequence in comparison with that of *A. muciniphila* Muc^T^ [55]. Strikingly, while the 16S rRNA sequences of strains H3, H4, H5 and H6 showed no mismatches with that of the type-strain Muc^T^, strains H1 and H2 showed three deviations. These were located at *E. coli* positions 183, 188 and 1285, and were predicted to be located in the variable loop structure of the 16S rRNA.

**Figure 1:**
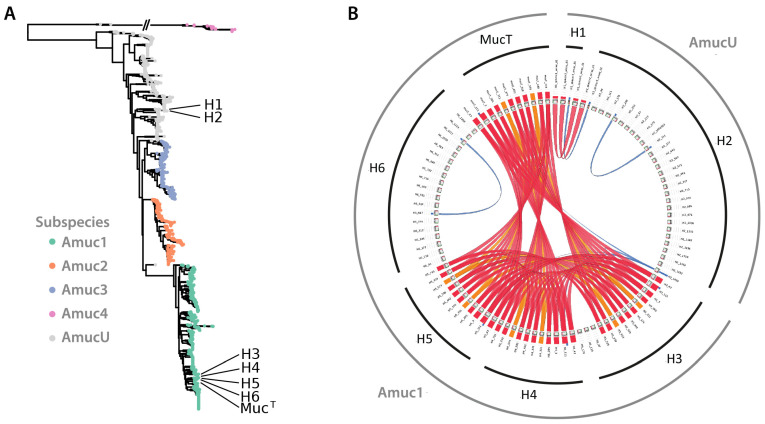
(**A**). Genome-based clustering of the new human isolates and the type-strain Muc^T^. The six new isolates (H1–H6) were positioned in the tree that was constructed earlier based on available (draft) genomes [22]. (**B**). Schematic representation of CRISPR repeats of the new human isolates and the type-strain Muc^T^**.** All CRISPR repeats are compared with each other. The red ribbons indicate a relatively high E-value (80% of max), orange indicates a lower E-value (max 60%), with blue depicting an E-value of maximum 20%. The newly isolated strains are visualized in the inner black circle. *A. muciniphila* subspecies are indicated in the outer grey circle.

The draft genome sequences of the new *A. muciniphila* strains were determined and used in comparative genome analysis with the genomes of a large set of human-associated *Akkermansia* species studied for their genomic diversity and ecology [22]. This resulted in the clustering of the human isolates in two different subspecies (Figure 1A). Strains isolated from the same subject clustered together (i.e., H3 with H4, and H5 with H6). All these strains cluster together in the Amuc1 subspecies, together with the type-strain Muc^T^. Strains H1 and H2 also clustered together but belonged to the AmucU class of unassigned subspecies of *A. muciniphila* (Figure 1A).

### 3.2. Growth Characteristics of the Six Akkermansia Isolates

All six new isolates showed optimal growth in mucin-based medium. Growth as measured by optical density (OD_600_ of 1.57 ± 0.07), specific growth rate (0.33 ± 0.04 h^−1^) and production of SCFAs was comparable for all isolates: acetate (12.96 ± 1.50 mM), propionate (6.97 ± 0.84 mM), succinate (1.82 ± 0.50 mM) and 1,2-propanediol (0.79 ± 0.10 mM) (Full data per strain can be found in Figure S1 and Table S2).

While the fermentation of mucin resulted in the formation of acetate, propionate and 1,2-propanediol, only acetate and propionate were formed after glucose fermentation. Weak growth was monitored on glucose, N-acetylglucosamine and fucose, but only after further supplementation with high amounts of tryptone (16 g/L). No growth was observed on N-acetylgalactosamine, maltose, mannose or lactose. The growth (both in yield and rate) of strains H1 and H2 in glucose-based medium with tryptone was significantly lower than that of the other strains, whilst the ratio of propionate:acetate production remained similar (Table S2).

### 3.3. Antibiotic Resistance Profiles of the Six A. muciniphila Isolates in Comparison with These of the Akkermansia Type-Strains

The resistance to antibiotics of the *A. muciniphila* strains was assessed by determining the Minimal Inhibitory Concentration (MIC) that allowed growth on plates. The MIC-values of the six isolates were compared to the *A. muciniphila* type strain Muc^T^ and the non-human isolated *A. glycaniphila* Pyt^T^ [20]. A total of fifteen antimicrobials were tested in the ANO2B plates, whilst sixteen different antibiotics were tested in the VetMIC plates. These antibiotics included those considered to be a basic requirement according to EFSA (European Food Safety Authority, Parma, Italy) guidelines [28]. Assessment of the MIC results of the control strains indicated that both procedures and plates worked accurately.

The MIC-values observed for the *A. muciniphila* type-strain and isolates were all very similar and slightly differed from those obtained for *A. glycaniphila* Pyt^T^. In general, levels were very comparable for all human isolates, and lower than those obtained for the *A. glycaniphila* Pyt^T^ strain (Tables S3 and S4). *A. glycaniphila* Pyt^T^ was found to be more sensitive to kanamycin and some other aminoglycosides.

Compared to the control strain *Bacteroides fragilis* ATCC25285, the *Akkermansia* strains presented lower MIC values for most tested antibiotics, with the exceptions of imipenem, cefoxitin and tetracycline. In the VetMIC-plates, the resistance of *Akkermansia* strains to aminoglycosides was higher than that of the control strain *Lacticaseibacillus paracasei* ATCC 334, likely due to the anaerobic metabolism of the *Akkermansia* strains. In spite of the presence of genes suggested to be involved in antibiotic resistance, such as β-lactamases (Amuc_0106 and Amuc_0183) [19], the resistance to β-lactam antibiotics was for the *Akkermansia* strains equal or just slightly higher compared to that of the *L. paracasei* strain in the VetMIC-plates (penicillin and ampicillin) and lower than that of the corresponding *B. fragilis* control strain in the ANO2 plates (penicillin, ampicillin, cefotetan, imipenem).

The high conservation of the antibiotic resistance patterns between *A. muciniphila* Muc^T^ and the novel isolates indicates that the observed antibiotic resistances are rather intrinsic than acquired, according to EFSA guidelines [28]. Therefore, none of the *A. muciniphila* strains seem to pose strain-specific risks in terms of their antibiotic resistance patterns nor a significant risk for the horizontal transfer of antibiotic resistance determinants.

### 3.4. Comparative Genomics Confirms That the New Isolates Belong to Two Phylogenetic Clusters

The genomes of the newly obtained *A. muciniphila* isolates were determined by Illumina sequencing and compared to that of the Muc^T^ type-strain [19] (Table 1). The strains showed comparable genome sizes, GC content and total number of genes. The average nucleotide identity (ANI) of the strains was found to be between 97.6–100%, which is above the proposed threshold of 97.6% for new species [56], confirming their classification as belonging to the species *A. muciniphila*. The BLAST similarity (using regions of >5 kb) of H3, H4, H5 and H6 showed a high similarity with Muc^T^ (>99.9) and a lower similarity for H1 and H2 (>97.62). Along the same lines, the related genomes of H1 and H2 showed the largest number of Single Nucleotide Polymorphisms (SNPs) with the genome of the type-strain Muc^T^. This contrasts to the genomes of the other isolates, which are highly similar to each other and the Muc^T^ genome (Table 1).

### 3.5. Evaluation of CRISPR-Cas Systems in Novel A. muciniphila Isolates

To assess the CRISPR-Cas systems predicted by the genomes of the *A. muciniphila* isolates, the presence of genes encoding Cas1 (*Amuc_2009*), Cas2 (*Amuc_2008*) and Cas9/Csn1 (*Amuc_2010*) was evaluated [57]. In total three different CRISPR arrays were identified, that all belonged to the same type (Superclass E) [48]. CRISPR targets were only found when the cut-off score was lowered from 20 to 0 and included mainly metagenomic datasets from gut DNA [50].

We analyzed the similarities within the spacers to identify potential shared history (Figure 1B). Strains H3, H4 and H5 and Muc^T^ were found to share most of their spacers, with H3 having acquired three extra spacers next to the leader sequence. Surprisingly, H6 seemed to have recruited an own set of spacers. Strains H1 and H2 shared three spacer sequences, with additional spacer sequences being detected in the complete genome of H2 (see below). Although these strains were not isolated from the same subject, they still shared three spacer sequences, confirming their high conservation deduced from the SNP analysis (Table 1). Strains H3 and H4, which were isolated from the same subject, shared all but one spacer sequence, indicating they are different, but highly related strains. In contrast, strains H5 and H6, which also were isolated from the same subject, did not share any spacers, suggesting that highly similar *A. muciniphila* strains exist within the intestinal tract of the same subject but apparently differ in their exposure to plasmids or viruses.

Alignment of CRISPR sequences further supported the breakdown of the isolates in two large groups (Figure 1B). The draft genomes, CRISPR-sequences and phylogenetic analyses all supported the presence of two groups within the isolated strains. Just like the *A. muciniphila* type-strain Muc^T^, the strains H3, H4, H5 and H6 belong to the Amuc1 cluster, whilst the other two strains H1 and H2 clustered in the AmucU cluster (see above) [22]. In the remainder of this work we mainly focus on the type-strain Muc^T^ as a representative of the Amuc1 cluster of isolates, as its full genome was determined [19] and H2 as a proxy for the two isolates H1 and H2 belonging to the cluster AmucU [22].

### 3.6. Comparative Genomics Shows the Presence of Inversions and Rearrangements between Genomes of Strains H2 and Muc^T^

**Figure 2:**
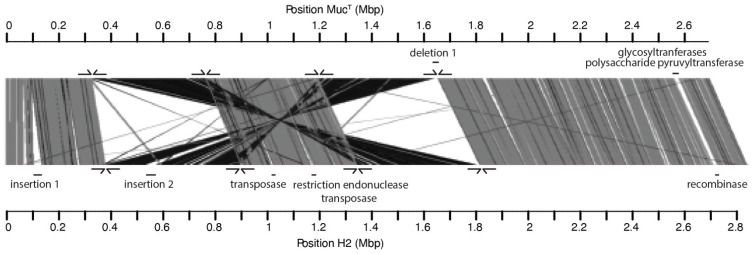
Comparison of the complete genomes of Muc^T^ and H2. Alignment of the genomes of Muc^T^ and H2 in ACT [58] and visualized in a linear way using the WebACT comparison tool [59]. (-) indicates non-homologous sequences. Arrows indicate primer-binding sites.

The complete genome of *A. muciniphila* strain H2 as representative of the subspecies harboring H2 and H1 was determined by single molecule sequencing (PacBio SMRT) and compared to the genome of *A. muciniphila* Muc^T^ as a representative of the Amuc1 cluster. Careful inspection of the two genomes revealed a number of large insertions in the genome of strain H2, which were not present in the Muc^T^ genome. Moreover, two major inversions and two rearrangements were observed, which is evident from the XY plot comparing the genomes (Figure S2). To study the distribution of these rearrangements, primers for the junctions were designed (Figure 2). These were used to confirm the presence of the same genomic rearrangements in both strains (H1 and H2) while the strains H3, H4, H5 and H6 had a similar genome arrangement as strain Muc^T^.

The complete genome of H2 was found to be 156 kb larger than the genome of type-strain Muc^T^. This additional DNA was mainly found in two large genomic insertions (Figure 2). The first genomic insertion was predicted to have a size of approximately 40 kb and included genes predicted to be encoding for an integrase, serine/threonine protein kinase, adenine-specific DNA methylase and multiple endonucleases. Moreover, the GC content of these regions (49%) was different compared to the rest of the genome (55%), indicative of a foreign DNA insertion. The second genomic insertion was slightly larger, with a size of approximately 50 kb, with genes coding for an integrase, head-to-tail joining protein (podovirus-type) and other phage-like proteins. The GC skew was opposite compared to its flanking regions in the chromosome, also indicative of a genomic insertion of a bacteriophage (remnant). Other, smaller insertions showed a similarly lowered GC/AT-ratio or opposite GC skew and included integrases and recombinases, pointing towards potential phage remnants. One additional deletion was observed where a glycosyltransferase and a polysaccharide pyruvyltransferase were found to be absent from the complete H2 genome (Figure 2).

In addition to the insertions and deletions, comparative analysis of the complete genomes of *A. muciniphila* strains Muc^T^ and H2 revealed 62,508 SNPs, that were found to be evenly distributed over the H2 genome, with the exception of nine intergenic regions, where the SNPs were found to be fifteen times more abundant than the average 2.2%, suggesting evolutionary pressure for conservation of the coding regions.

### 3.7. Glycan-Degradation Capacity Is Conserved across All Isolates, but H2

When comparing the predicted glycan degradation CAZyome of all newly isolated *A. muciniphila* strains to that of the type-strain Muc^T^ and *A. glycaniphila* Pyt^T^, it became apparent that especially the deduced glycan degradation potential of the strain H2 is significantly lower compared to the other isolates (Table 2). Strain H2 is predicted to have a reduced amount of glycoside hydrolases of many classes, with enzymes of certain families even completely lacking. Hence, it seems that strain H2 has lost the capacity to degrade several carbohydrates, which is strikingly not (or only to a minor extent) the case for strain H1. The genomes of strains H1 and H2 lacked the complete coding sequences for four glycan-degrading enzymes, including *Amuc_0623* (a very distantly relative of sialidases), *Amuc_1666* (β-galactosidase), *Amuc_2164* (β-N-acetylglucosaminidase) and one of the two α-L-fucosidases present in the Muc^T^ strain (*Amuc_0146*).

The predicted glycan degradation enzymes in the human isolates of the Amuc1 subspecies (H3, H4, H5, H6) strongly aligned to each other and the ones predicted in the type-strain Muc^T^ (Table 2). When zooming in on the predicted polysaccharide lyase capacity of all strains, no differences could be discerned between the isolates and Muc^T^ since all strains are predicted to harbor a representative of the PL38 glucuronan lyase (*Amuc_0778*).

Irrespective of the lack of certain glycoside hydrolases in strain H2 compared to the other human isolates, all newly isolated strains are able to grow to a similar extent on mucin (see earlier, Figure S1). This suggests that the mucin-degrading capacity of all new isolates is intact.

The adaptation of the glycoside hydrolase potential of the newly isolated human strains also becomes apparent when comparing the distribution of CAZymes over the various families with the non-human *A. glycaniphila* Pyt^T^. This latter strain has a more varied and higher glycoside hydrolase profile compared to the human isolates and the type-strain Muc^T^ (Table 2). This is also reflected in the presence of extra polysaccharide lyases compared to the human isolates: apart from the PL38 enzyme also present in the human isolates, also a PL8 chondroitin and PL12 heparin sulfate lyase are found in the *A. glycaniphila* CAZyme portfolio.

The difference in the predicted CAZyme profiles between the human isolates and the python-isolated *A. glycaniphila* Pyt^T^ likely reflects their different ecological niches, with the human isolates adapted to mucin degradation.

### 3.8. Comparative Proteome Analysis of A. muciniphila Strain H2 and A. Glycaniphila

Proteome characterizations have been used to show the adaptation of the *A. muciniphila* type-strain Muc^T^ to various growth media [60,61]. Here we focused on the proteome of *A. muciniphila* strain H2 and that of *A. glycaniphila* Pyt^T^ as these show the largest difference in their glycoside hydrolase portfolio. These strains were grown on a synthetic medium and proteome analysis identified over 600 proteins in each culture (Supplementary File). Of note, the proteome of *A. muciniphila* strain H2 (in total 610 proteins detected) included only two glycoside hydrolases, in stark contrast with the total of 18 found to be produced by *A. glycaniphila* Pyt^T^ (total of 729 proteins detected). When focusing on the top 100 most abundantly produced proteins, it was assessed whether these had a homologue in the *A. muciniphila* type-strain Muc^T^. For *A. muciniphila* strain H2, a total of 79 proteins could be mapped to the Muc^T^ proteome, whilst 16 were mapped to an UniParc ID, and for 8 proteins no hits could be found. Similar numbers were recorded for the proteome of *A. glycaniphila* strain Pyt^T^: 80 Muc^T^ homologues, 12 UniParc IDs and 8 proteins with no hits. When comparing these top 100 of the strongest expressed proteins in the Pyt^T^ and H2 isolate proteome samples, we found 42 proteins in common. Many of these are the highly expressed ribosomal proteins but also present in the proteomes of strains Pyt^T^ and H2 are the Amuc_1098 and Amuc_1099 proteins, which are in the same operon as the key functional Amuc_1100 protein in the Muc^T^ type strain [10,11,14] (in yellow in Supplementary File). Amongst the most abundant proteins we detected many glycosylation-related genes involved in glycan metabolism (indicated in blue and in a separate tab in Supplementary File).

## 4. Discussion

By using enrichments on mucin as sole carbon, energy and nitrogen source, we isolated six new *A. muciniphila* strains from healthy human subjects. Based on their genomic, 16S rRNA and CRISPR sequences, the strains were found to be containing unique CRISPR sequences and could be clustered into two different groups of *A. muciniphila*: strains H1 and H2 are amongst an unassigned cluster of strains, termed here AmucU, whilst H3, H4, H5 and H6, cluster together with the type-strain Muc^T^ into the Amuc1 cluster (Figure 1) [22]. However, their physiological growth parameters were very similar to that of type-strain Muc^T^, including growth rate and yield as well as substrate use and production of propionate and acetate. Only minor differences were observed for the AmucU cluster strains H1 and H2 that showed some growth delay on non-mucus medium as compared to the other isolates (Figure S1). Noteworthy, several strains (H3 and H4 as well as H5 and H6) were isolated from a fecal donation from a single healthy subject (Table S1), suggesting that simultaneous occurrence of multiple strains may occur regularly.

The complete genome sequence of strain H2 as a representative of the AmucU cluster was determined by single molecule sequencing and was found to share a high ANI (97.6%) with that of the Amuc1-cluster the type-strain Muc^T^ (Table 1) with SNPs that were distributed equally all over the genome. Further comparison of these genomes revealed two large genome 200-kb rearrangements and inversions. PCR-based analysis confirmed these to be present in all other isolates belonging to either of both subspecies. The same genomic arrangements with conserved insertion sites were shared between isolates belonging to the same subspecies. This finding and the high ANI of all genomes (Table 1) suggest a common ancestor of the strains, in spite of large genomic rearrangements.

The six genomes of the new isolated *A. muciniphila* strains are all similar in size to the one of Muc^T^ [19], and with 2.6–2.8 Mb smaller than the mean average genome size of 3.9 Mb (range 2.8–5.8 Mb) estimated for GI-tract colonizers [62]. Other mucin-degrading bacteria that are not solely dedicated to mucin utilization, generally have larger genome sizes, such as *Bacteroides thetaiotaomicron* VPI-5482 (6.3 Mb), *Ruminococcus gnavus* AGR2154 (3.72 Mb) or *B. fragilis* YCH46 (5.3 Mb). It has been proposed that bacteria that are adjusted to a specific niche, have a reduced genome size [63]. This becomes apparent when considering the genome size of the intracellular pathogens *Mycoplasma pneumoniae* M129 (0.82 Mb) and *Helicobacter pylori* 26,695 (1.67 Mb). Hence, it is tempting to assume that also a beneficial species such as *A. muciniphila* has a reduced genome size as a result of its specialization to its mucosal niche. This is also supported by the observation that all known *A. muciniphila* strains have an almost identical, but very narrow substrate utilization range. Supporting this hypothesis is the fact that the genome of the *A. glycaniphila* type-strain Pyt^T^ has a slightly larger size of 3.0 Mb [21]. In line, this *A. glycaniphila* strain was found to have a larger set of predicted glycoside hydrolases than any of the tested other *A. muciniphila* strains (Table 2), suggestive of a broader glycan-degradation potential, and this was confirmed by its proteomic characterization (Supplementary File).

There is very high genomic similarity (>97%) between the six new *A. muciniphila* isolates and the type-strain Muc^T^ type (see Table 1). This low genomic divergence could be a further characteristic of its adaptation to the mucus layer of the healthy human GI tract. This is reflected in a strong conservation of the glycoside hydrolase portfolio of all isolated strains. As mucin is not a diet-related energy source, *A. muciniphila* does not need to adapt its genetic potential to fluctuating energy sources. This in contrast to the hundreds of species belonging to the two most abundant phyla in the GI tract, i.e., Firmicutes and Bacteroidetes [64]. The strong conservation of the mucin-degrading genes of *A. muciniphila* is further confirmed in recent analysis of Korean, US and Chinese isolates, where retention of mucin-degradation was identified as one of the strong evolutionary forces affecting *A. muciniphila* strains [65]. Interestingly, this was demonstrated for *A. muciniphila* strains isolated from various mammals, which also showcased low genomic divergence and a strong specialization in mucus colonization and degradation [66].

A special case is the newly isolated *A. muciniphila* strain H2 strain that is predicted to have a reduction in the number and variety of glycoside hydrolases (Table 2). This offers leads towards identifying the minimal amount of enzymes and proteins necessary to degrade mucin, as this strain showed no growth delays on mucin as its sole carbon and nitrogen source (Figure S1). Proteomic analysis of *A. muciniphila* strain H2 confirmed the low amount of produced glycoside hydrolases as only one could be identified in the over 600 abundant proteins. In contrast, *A. glycaniphila* strain Pyt^T^ produced 16 glycoside hydrolases under the same experimental conditions. Both species abundantly produced the outer-membrane secretin PilQ, involved in the production of type IV pilus [60]. Similarly, both species also produced, although at a reduced level, the heat-stable outer-membrane protein Amuc_1100, which in the type-strain *A. muciniphila* type-strain Muc^T^ has found to signal to the Toll-Like Receptor 2 (TLR2) and induce barrier function in human cell lines and mice [10,11,14].

All isolated *A. muciniphila* strains produced propionate but none was capable of vitamin B12 synthesis (the cofactor for the methyl-malonyl CoA synthase reaction) in contrast to *A. muciniphila* isolates of other phylogenetic clusters and the *A. glycaniphila* type-strain Pyt^T^, testifying for the loss of this cofactor production capacity in the Amuc1 and AmucU clades [22].

Finally, we also determined the antimicrobial resistance profiles of these novel isolates and compare these with the *A. muciniphila* and *A. glycaniphila* type-strains. The results demonstrated the absence of problematic antibiotic resistances, supporting the genomic analysis that showed no transferable antibiotic resistance genes that are found in some *A. muciniphila* isolates [22]. Moreover, the high conservation of the resistance patterns between *A. muciniphila* Muc^T^ and all new isolates indicates that the antibiotic resistances are intrinsic, rather than acquired (Tables S3 and S4) [28]. This is of key importance, as it illustrates that neither of the *A. muciniphila* strains poses strain-specific risks in terms of antibiotic resistance patterns, nor significant risks towards the horizontal transfer of antibiotic resistance determinants. These findings further support the evaluation and application of *A. muciniphila* as a next-generation beneficial microorganism [11]. It will be of interest to further perform a comparative evaluation of the impact of the novel isolated strains in the context of obesity, inflammation and other pathologies where the *A. muciniphila* Muc^T^ strain has shown convincing promise [11].

## 5. Conclusions

Here, we present six novel human isolates of *A. muciniphila* originating from the GI tract of healthy humans. The strains belong to two different clusters (Amuc1 and AmucU) of *A. muciniphila* and differ slightly due to genomic rearrangements, although there is little divergence in their coding capacity, genomic and physiological properties. All isolates show adaptation to their mucosal niche, sharing conserved glycan-degrading capacities, although strain *A. muciniphila* H2, belonging to the AmucU cluster, displayed a significantly reduced CAZyme diversity. The isolation and comparative genomic and physiological characterization of these novel isolates further opens avenues towards the application of *A. muciniphila* strains as next-generation beneficial microorganisms.

## Figures and Tables

**Table 1:** Sequence characteristics of the new *A. muciniphila* isolates and comparison to genome of the type-strain Muc^T^ (ANI, BLAST similarity and SNP).

Strain	Coverage	Contigs	Genome Size (Mbp)	GC Content (%)	Total Gene Count	ANI (%)	BLAST Similarity (>5 kb, %)	SNP
Muc^T^	NA	1	2.7	55.76	2178	100.00	100.00	0
H1	60	34	2.8	55.04	2350	97.67	97.20	62,763
H2	100	1	2.8	55.35	2348	97.62	97.16	62,508
H3	120	30	2.7	56.05	2243	99.99	100.00	3
H4	150	34	2.6	56.11	2204	99.99	100.00	3
H5	150	18	2.5	55.80	2055	100.00	99.97	25
H6	140	22	2.7	55.06	2208	99.99	99.88	27

NA: not applicable, ANI: Average Nucleotide Identity, SNP: Single Nucleotide Polymorphism.

**Table 2:** Family classification of predicted glycoside hydrolases (GH) in the human isolates of *Akkermansia muciniphila.* The non-human isolate *A. glycaniphila* strain Pyt^T^ was also included in the analysis.

CAZy Family	GH 2	GH 3	GH 13	GH 16	GH 18	GH 20	GH 27	GH 29	GH 31	GH 33	GH 35	GH 36	GH 43	GH 57	GH 63	GH 73	GH 77	GH 84	GH 89	GH 95	GH 97	GH 105	GH 109	GH 110	GH 115	GH 123	GH 163
**Muc^T^**	6	1	3	3	1	11	1	4	2	3	2	3	2	1	1	0	1	1	2	2	1	1	2	2	0	1	0
**H1**	5	1	3	3	0	11	1	4	2	3	2	3	1	1	1	0	1	2	2	2	1	1	2	2	0	1	0
**H2**	3	0	2	1	0	6	1	2	1	2	1	1	1	1	1	0	1	0	2	1	0	0	2	0	0	0	0
**H3**	6	1	3	3	1	11	1	4	2	3	2	3	2	1	1	0	1	1	2	2	1	1	2	2	0	1	0
**H4**	6	1	3	3	1	11	1	4	2	3	2	3	2	1	1	0	1	1	2	2	1	1	2	2	0	1	0
**H5**	6	1	3	3	1	11	1	4	2	3	2	3	2	1	1	0	1	1	2	2	1	1	2	2	0	1	0
**H6**	6	1	3	3	1	11	1	4	2	3	2	3	2	1	1	0	1	1	2	2	1	1	2	2	0	1	0
**Pyt^T^**	10	3	2	2	3	15	1	7	2	6	1	6	1	1	1	2	1	1	3	3	0	0	1	2	1	1	1

## Data Availability

All data are included in the manuscript as supplementary information and accession numbers of all genomes are included.

## Supplementary Materials

The following supporting information can be downloaded at: https://www.mdpi.com/article/10.3390/microorganisms10081605/s1, Figure S1: Growth curves on mucin and glucose of newly isolated strains and Muc^T^; Figure S2: XY plot of H2 versus Muc^T^; Table S1: Host characteristics and accession numbers of the *A. muciniphila* human isolates and the Muc^T^ type strain; Table S2: Growth characteristics on mucin and glucose of newly isolated strains and Muc^T^, Table S3: Minimum Inhibitory Concentrations (μg/mL) of Akkermansia strains and isolates in GAM medium in ANNO2B plates, Table S4: Minimum Inhibitory Concentrations (μg/mL) of Akkermansia strains and isolates grown in GAM medium on VETMIC Lact-1 and Lact-2 plates, File S1—Proteomics.
