# Supplementary material for: Comparative Genomics and Physiology of Akkermansia muciniphila Isolates from Human Intestine Reveal Specialized Mucosal Adaptation
Source: Microorganisms. 2022 Aug 9;10(8):1605. doi: 10.3390/microorganisms10081605 (PMC9415379; doi:10.3390/microorganisms10081605)
Supplement: Supplementary file 1 [file microorganisms-10-01605-s001.zip › microorganisms-1822173-supplementary.pdf]

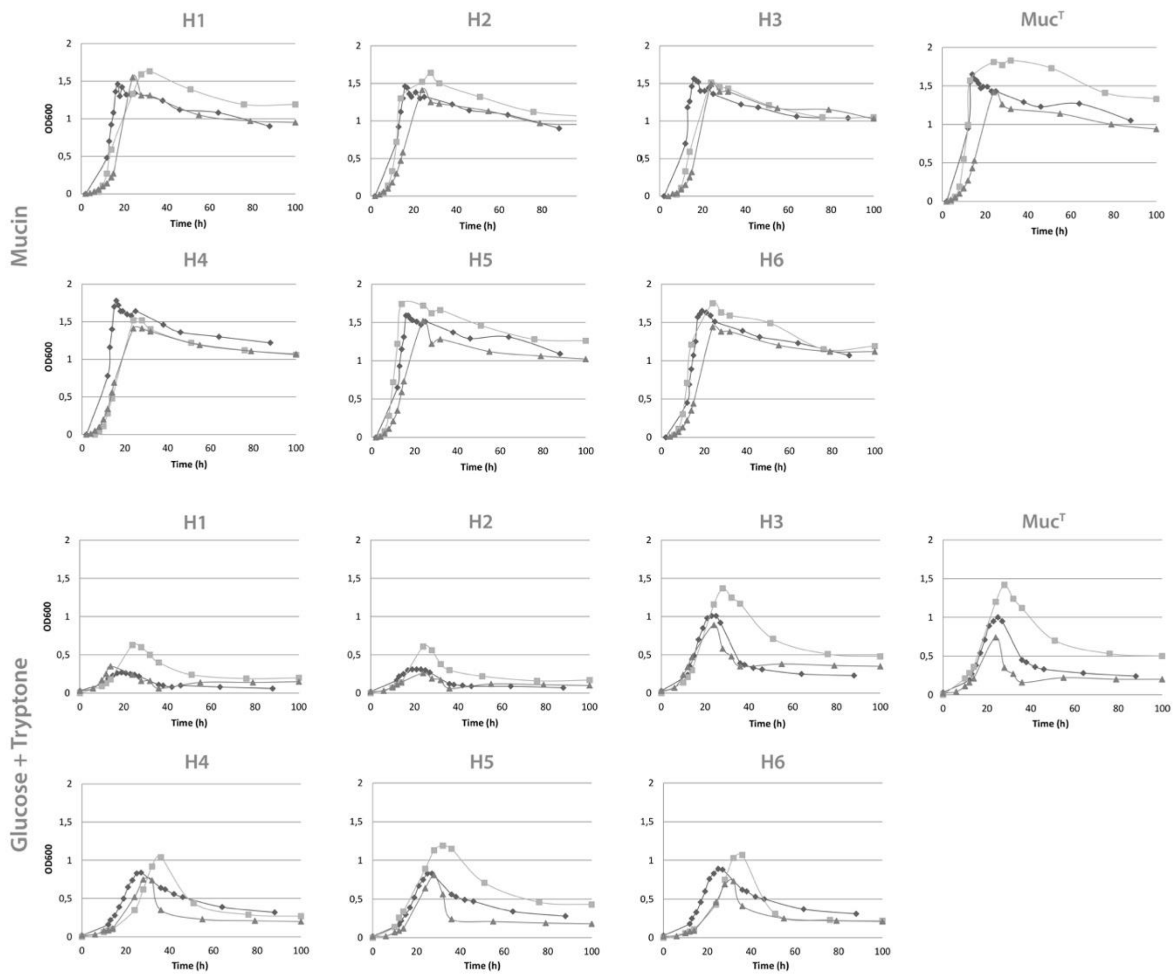

Figure S1. Growth curves on mucin and glucose of newly isolated strains and Muc^T. Growth curves were repeated three times. All individual curves are depicted in different colors.

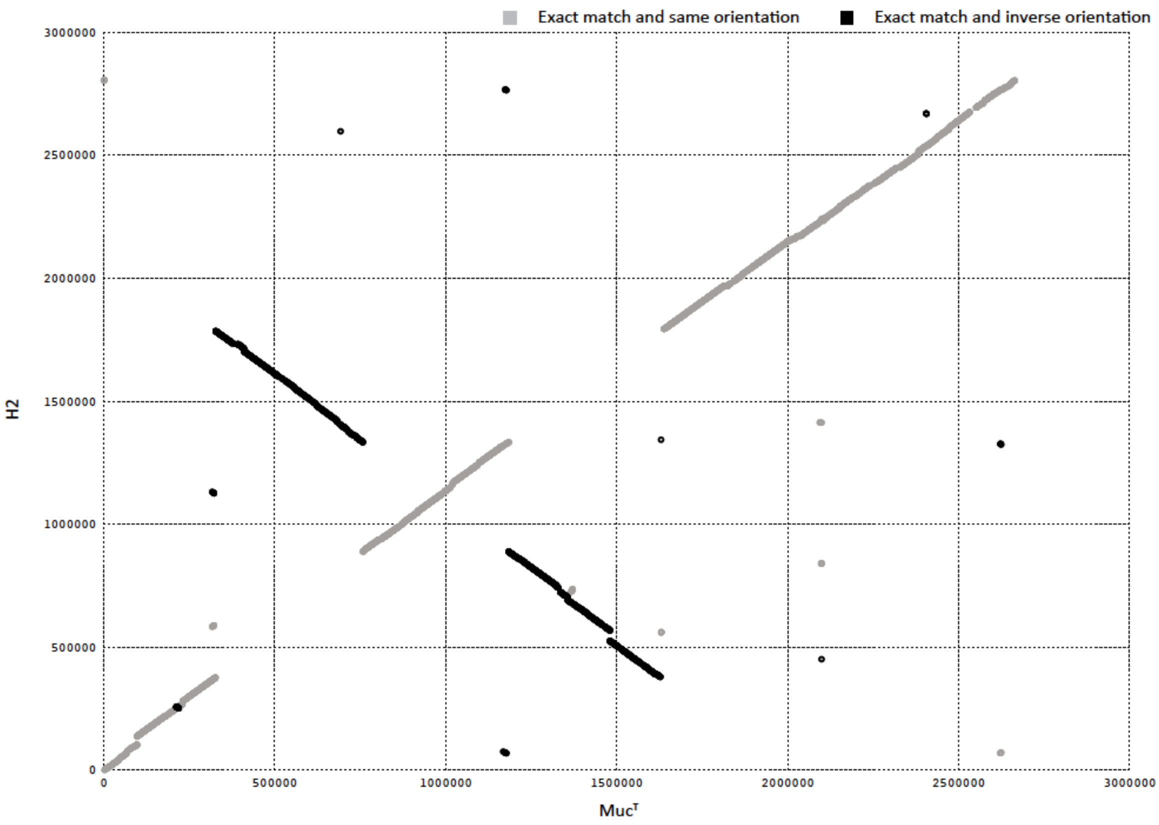

Figure S2. **XY plot of H2 versus Muc^T.** A sequence comparison between H2 and Muc^T was performed using the MUMMER package with a minimum length of 100 bp exact match. Grey dots indicate exact match and same orientation. Black dots indicate exact match and inverse orientation.

Table S1. Host characteristics & Accession numbers of the *Akkermansia muciniphila* human isolates and the Muc^T type strain.

Strain	Accession number	Nationality	Age	Gender
Muc^T	NC_010655	France	30	Female
H1	SAMN03278369	Belgium	33	Male
H2	SAMN03278370	Belgium	32	Male
H3, H4	SAMN03278371, SAMN03278372	Belgium	24	Male
H5, H6	SAMN03278373, SAMN03278374	Netherlands	35	Female

Table S2. Growth characteristics on mucin and glucose of newly isolated strains and Muc^T. Average (n=3) and standard deviation (SD) are depicted. When growth yield was significantly different compared to Muc^T using the student t-test (p<0.05) an asterisk is included.

Mucin

Strain	Growth rate (SD)	Growth yield (SD)	SCFA production				Ratio acetate/(propionate + succinate) (SD)	Ratio acetate/propionate (SD)
			Acetate (SD)	Propionate (SD)	1,2 Propanediol (SD)	Succinate (SD)		
H1	0.35 (0.14)	1.55 (0.09)	12.41 (1.09)	6.42 (0.58)	0.92 (0.11)	2.09 (0.78)	1.46 (0.17)	1.94 (0.22)
H2	0.28 (0.08)	1.50 (0.12)	11.90 (1.07)	5.50 (0.67)	0.80 (0.15)	2.11 (0.57)	1.54 (0.12)	2.17 (0.19)
H3	0.39 (0.15)	1.52 (0.04)	12.82 (1.74)	7.52 (0.30)	0.75 (0.13)	1.23 (1.36)	1.48 (0.16)	1.71 (0.23)
H4	0.33 (0.07)	1.57 (0.19)	12.72 (1.60)	7.24 (0.08)	0.73 (0.17)	1.67 (1.04)	1.43 (0.14)	1.76 (0.24)
H5	0.29 (0.01)	1.62 (0.11)	16.28 (4.07)	8.17 (1.11)	0.90 (0.12)	2.62 (1.74)	1.52 (0.20)	1.98 (0.33)
H6	0.34 (0.07)	1.61 (0.16)	12.52 (0.37)	6.94 (0.88)	0.79 (0.05)	1.47 (1.46)	1.50 (0.21)	1.83 (0.27)
Muc^T	0.31 (0.10)	1.63 (0.21)	12.09 (5.26)	7.00 (3.89)	0.64 (0.24)	1.43 (0.11)	1.46 (0.11)	1.88 (0.46)

Glucose + Tryptone

Strain	Growth rate (SD)	Growth yield (SD)	Glucose consumption (SD)	SCFA production			Ratio acetate/(propionate + succinate) (SD)	Ratio acetate/propionate (SD)
				Acetate (SD)	Propionate (SD)	Succinate (SD)		
H1	0.09 (0.04)	0.42 (0.19)*	7.34 (3.05)	3.99 (2.09)	5.02 (1.72)	0.50 (0.43)	0.69 (0.12)	0.76 (0.17)
H2	0.11 (0.05)	0.39 (0.19)*	7.49 (2.81)	4.79 (1.22)	4.38 (2.16)	1.33 (0.05)	0.87 (0.11)	1.17 (0.25)
H3	0.14 (0.01)	1.09 (0.25)	6.60 (3.26)	7.62 (0.94)	11.10 (1.65)	1.04 (0.44)	0.64 (0.16)	0.70 (0.17)
H4	0.14 (0.02)	0.88 (0.15)	12.93 (2.95)	4.86 (1.04)	8.01 (2.57)	0.65 (0.58)	0.59 (0.17)	0.63 (0.20)
H5	0.15 (0.03)	0.94 (0.21)	8.32 (2.66)	5.62 (1.64)	8.71 (2.15)	0.94 (0.63)	0.59 (0.13)	0.65 (0.17)
H6	0.15 (0.02)	0.90 (0.17)	9.68 (1.95)	5.15 (1.08)	8.30 (2.48)	0.50 (0.49)	0.61 (0.17)	0.64 (0.17)
Muc^T	0.14 (0.02)	1.05 (0.34)	12.89 (3.89)	6.73 (1.61)	9.03 (5.06)	0.76 (0.45)	0.94 (0.70)	1.05 (0.81)

Table S3. Minimum Inhibitory Concentrations (μg/mL) of Akkermansia strains and isolates in GAM medium in ANNO2B plates

Antibiotic	Muc[T]	H1	H2	H3	H5	H6	Pyt[T]	*B. fragilis* ATCC25285
Ampicillin/sulbactam 2:1 ratio	≤0.5/0.25	≤0.5/0.25	≤0.5/0.25	≤0.5/0.25	≤0.5/0.25	≤0.5/0.25	1/0.5	2/1
Amoxicillin/clavulanic acid 2:1 ratio	≤0.5/0.25	≤0.5/0.25	≤0.5/0.25	≤0.5/0.25	≤0.5/0.25	≤0.5/0.25	≤0.5/0.25	1/0.5
Cefotetan	≤4	≤4	8	8	8	8	64	8
Penicillin	1	1	1	1	1	0.5	2	4
Imipenem	1	0.5	0.5	1	1	2	4	0.5
Meropenem	≤0.5	≤0.5	≤0.5	≤0.5	≤0.5	≤0.5	≤0.5	0.5
Clindamycin	≤0.25	≤0.25	≤0.25	≤0.25	≤0.25	≤0.25	≤0.25	0.5
Cefoxitin	>32	>32	16	32	>32	>32	>32	8
Metronidazole	≤0.5	1	1	1	1	1	1	4
Chloramphenicol	4	≤2	4	≤2	≤2	4	≤2	8
Ampicillin	≤0.5	1	1	1	1	1	8	>16
Piperacillin	≤4	≤4	≤4	≤4	≤4	≤4	32	16
Tetracycline	8	8	4	2	2	4	4	0.5
Mezlocillin	≤4	≤4	≤4	≤4	≤4	≤4	16	32
Piperacillin/tazobactam constant 4	≤0.25/4	≤0.25/4	≤0.25/4	≤0.25/4	≤0.25/4	≤0.25/4	≤0.25/4	8/4

Table S4. Minimum Inhibitory Concentrations (μg/mL) of Akkermansia strains and isolates grown in GAM medium on VETMIC Lact-1 and Lact-2 plates

Antibiotic	Muc^T	H1	H2	H3	H5	H6	Pyt^T	*L. paracasei* ATCC334
Gentamicin	>128	>128	>128	>128	>128	>128	64	1
Kanamycin	>512	>512	>512	>512	>512	>512	128	32
Streptomycin	>128	>128	>128	>128	>128	>128	>128	16
Neomycin	>128	>128	>128	>128	>128	>128	128	2
Tetracycline	2	2	2	1	1	1	1	4
Erythomycin	>4	>4	>4	>4	>4	>4	4	0.25
Clindamycin	0.12	0.12	0.06	0.12	0.12	0.12	0.12	0.12
Chloramphenicol	1	1	2	1	1	1	0.5	8
Ampicillin	1	1	1	1	1	1	8	1
Penicillin	1	1	1	1	1	1	4	0.5
Vancomycin	>64	>64	>64	>64	>64	>64	64	>64
Quinupristin-dalfopristin	>4	>4	>4	>4	>4	>4	>4	1
Linezolid	0.5	0.5	0.5	0.5	0.25	0.5	0.25	4
Trimethoprim	2	1	1	1	1	2	8	>64
Cirpofloxacin	>64	>64	>64	>64	>64	>64	64	2
Rifampicin	1	1	1	1	1	1	0.5	0.5
